# Editorial: Cell Surface Proteins of Gram-Positive Pathogenic Bacteria

**DOI:** 10.3389/fmicb.2021.681880

**Published:** 2021-05-21

**Authors:** Magnus Hook, Timothy J. Foster

**Affiliations:** ^1^Center for Infectious and Inflammatory Diseases, Institute of Biosciences and Technology, Texas A&M Health Science Center, Houston, TX, United States; ^2^Microbiology Department, Trinity College Dublin, Dublin, Ireland

**Keywords:** surface proteins, adhesion, biofilm, immune evasion, iron acquisition, pili, S-layer

The surfaces of Gram-positive bacterial cells are decorated with a diverse array of proteins including a large group that are covalently anchored to peptidoglycan by sortases. Several types of sortase-anchored proteins can be differentiated on the basis of their structures. Notable examples reviewed in this special topic include MSCRAMMs (Arora et al.; Foster; Speziale and Pietrocola), serine rich repeat proteins (Chan et al.), antigen I/II proteins (Manzer et al.), NEAT motif containing proteins (Ellis-Guardiola et al.) and streptococcal fimbriae/pili (Ness and Hilleringmann; Nakata and Kreikemeyer).

Many proteins that are located of the surface of Gram-positive pathogens and not anchored covalently. *Clostridium difficile* and *Bacillus anthracis* are exemplars of bacteria whose outer surfaces comprise crystalline arrays of S layer proteins that are attached non-covalently to cell wall polysaccharides (Ravi and Fioravanti). Lipoproteins are anchored to the outer surface of the cytoplasmic membrane via membrane lipids (Nguyen et al.). Other non-covalently anchored surface proteins including moonlighting cytoplasmic proteins and extracellular vesicles are not reviewed here.

Some articles review specific protein types drawn from a single species for example fibronectin binding proteins (FnBP) of *Staphylococcus aureus* (Speziale and Pietrocola), surface proteins of *Staphylococcus epidermidis* (Foster) and pili of *Streptococcus pneumoniae and Streptococcus pyogenes* (Nakata and Kreikemeyer; Ness and Hilleringmann). Others describe protein families spanning different species/genera including proteins related to the biofilm associated protein BAP of *S. aureus* (Valle et al.), polymer adhesion domain containing proteins (Järvå et al.) and collagen binding proteins (Arora et al.).

An emerging theme is that a single protein can often carry out multiple functions. Proteins that are exposed on the surface of bacterial cells are in direct contact with the host and are subjected to selective pressure to perform functions related to colonization of host tissues and evasion of host defenses. The repertoire of proteins is limited so many have evolved to adopt multiple roles. Amino acid sequence variation can help explain niche and host specialization and why some murine models of infection are limited (Pickering and Fitzgerald).

The defining structural feature of MSCRAMM family is two adjacent IgG-like folded domains that bind ligands by the dock lock latch (DLL) or collagen hug mechanisms (Arora et al.; Foster; Speziale and Pietrocola). An important recent discovery is that binding of MSCRAMMs to their ligands is strengthened by shear forces to the extent that the force required for separation is equivalent to that required to break a covalent bond (Dufrêne and Viljoen). This is reminiscent of catch bonds of pili of uropathogenic *Escherichia coli* binding the urinary tract epithelium.

The N-terminal A domains of FnBPs of *S. aureus* are archetypal MSCRAMMs that bind fibrinogen by DLL in a shear force-enhanced manner (Speziale and Pietrocola; Dufrêne and Viljoen). The A domain also binds plasminogen and histones by non-DLL mechanisms and engages in homophilic interactions that promote biofilm formation. These ancillary functions contribute to pathogenesis and immune evasion. The C-terminal repeat region forms an unfolded flexible stalk that engages fibronectin by a tandem β-zipper mechanism.

The ability to bind collagens is an important feature of many Gram-positive bacterial pathogens. There are two well-known structural classes of collagen binding protein namely the MSCRAMM proteins related to CNA of *S*. aureus that bind collagens by the shear force-enhanced collagen hug mechanism and a subset of M proteins of *S. pyogenes* including those that carry the peptide associated with rheumatic fever in their N-terminal hypervariable domain (Arora et al.). There are three other less well-defined groups including the V region PAD of streptococcal antigen I/II proteins (Manzer et al.).

A structural motif that is widespread among surface proteins has herein been named the polymer adhesin domain (PAD) (Järvå et al.). PADs have been classified into groups based on structural similarities and functions. PADs can bind a diverse array of carbohydrate ligands such as glucans and dextrans, lipoteichoic acids and DNA. They can promote adhesion, cell aggregation, and plasmid conjugation. The ligand binding domain V of the antigen I/ll proteins of oral streptococci are class II PADs that binds protein ligands such as collagen and fibrinogen, promotes adhesion to the surface of epithelial and endothelial cells, and stimulates cellular aggregation and biofilm formation (Manzer et al.).

Surface proteins are involved in both the primary attachment and accumulation phases of biofilm formation (Foster; Speziale and Pietrocola). It has recently been recognized that the cell-cell aggregation and the accumulation phases involve surface proteins forming amyloid fibers in the biofilm matrix. This occurs with the BAP protein of *S. aureus* (Valle et al.), the accumulation associated protein Aap of *S. epidermidis* (Foster) and the SraP protein of *Streptococcus mutans* (Chan et al.).

Some Gram-positive pathogens express filamentous surface appendages called fimbriae/pili that are assembled and anchored to the cell surface by sortases. As pili are extruded from the bacterial cell subunits are covalently linked to each other by sortase-catalyzed isopeptide bonds. Structural analysis of pilins revealed intramolecular isopeptide bonds. P1 pili of *S. pneumoniae* bind a number of host proteins including collagens, they promote biofilm formation, bind receptors on host cells and promote passage of bacteria across the blood-brain barrier in the pathogenesis of meningitis (Ness and Hilleringmann).

Antigenic differences in *S. pyogenes* pili are responsible for the T serotyping scheme. These diverse pili can be categorized into nine different forms based upon heterogeneity of the encoding loci and sequence differences (Nakata and Kreikemeyer). Pili contribute to tropism for the skin or the nasopharynx. This is supported by enhanced expression at temperatures below 37°C that occur at these superficial niches. A novel feature of one of the minor pilins is to harpoon host ligands by formation of covalent thioester bonds.

Several species of streptococci express large serine rich repeat proteins that are heavily glycosylated by cognate glycosyltransferases (Chan et al.). The variable binding region (BR) is sandwiched between serine rich regions at the N-terminus and the longer C-terminal region. The BR of the Srr1 protein of *Streptococcus agalactiae* comprises an MSCRAMM motif that binds fibrinogen and keratin by DLL and contributes to adhesion to endothelial cells and vaginal epithelial cells promoting endocarditis and vaginal colonization, respectively. Both Srr1/2 and PsrP of *S. pneumoniae* play important roles in the transition from the commensal carriage state to invasive infection.

Pathogenic bacteria need a source of iron when growing in the host in order to overcome nutritional immunity. *S. aureus and Staphylococcus lugdunensis* encode iron regulated surface determinant (Isd) systems characterized by surface anchored proteins with NEAT motifs (Ellis-Guardiola et al.). These bind hemoglobin (Hb) and extract bound heme by forcing conformational changes in Hb. It is postulated that Isd proteins form protein wires that relay heme from the cell surface though the peptidoglycan to the membrane transporter.

The special topic also considers some non-covalently anchored surface proteins. All Gram-positive pathogens express lipoproteins that perform a multiplicity of functions (Nguyen et al.). The protein parts assist in nutrient acquisition, have chaperone activity, promote invasion of host cells and conjugation. When released from the bacterium the lipid moiety activates TLR2 pathways in host cells triggering inflammatory responses.

Recent structural studies provide insights into the S-layer and S-layer associated proteins (Ravi and Fioravanti). This includes localizing conserved domains involved in S-layer assembly and folds that promote attachment to cell surface polysaccharides. S-layer proteins are excellent candidates for future translational studies in diagnostics, therapeutics and vaccines.

Surface proteins can confer specialization for different hosts as well as colonization of specific niches within the host (Pickering and Fitzgerald). Staphylococcal IsdB proteins have a higher affinity for human hemoglobin than for the mouse protein which compromizes the study of virulence in murine infection models. Host specificity is also conferred by MSCRAMMs binding to the variable α-chain of fibrinogen (Fg). The ClfB protein of *S. aureus* binds to a human specific sequence and has very low affinity for canine Fg. In contrast the SpsL protein of *Staphylococcus pseudintermedius* binds strongly to the canine Fg α-chain and weakly to the human version. By manipulating a key adhesin/invasin-ligand interaction it is possible to create infection models that more closely resemble the human disease. Internalin A (InlA) binding to E-cadherin is key to invasion of the intestinal epithelium by *Listeria monocytogenes*. Transgenic mice expressing human E cadherin in the intestinal epithelia could be infected orally whereas wild-type mice are resistant. Conversely by altering residues in InlA to strengthen binding to murine E-cadherin it was possible to “murinize” the pathogen.

In conclusion this special topic provides a comprehensive and up-to-date summary of many important aspects of proteins that are located on the surfaces of Gram-positive bacterial pathogens and provides a useful starting point for anyone who wishes to obtain an overview of this important topic.

## Author Contributions

All authors listed have made a substantial, direct and intellectual contribution to the work, and approved it for publication.

## Conflict of Interest

The authors declare that the research was conducted in the absence of any commercial or financial relationships that could be construed as a potential conflict of interest.

